# Professional Interventions That Facilitate 12-Step Self-Help Group Involvement

**Published:** 1999

**Authors:** Keith Humphreys

**Affiliations:** Keith Humphreys, Ph.D., is a research psychologist at the Center for Health Care Evaluation, Veterans Affairs Palo Alto Health Care System, and an assistant research professor of psychiatry and behavioral sciences at Stanford University School of Medicine, Stanford, CA

**Keywords:** twelve step program, intervention, treatment outcome, cognitive therapy, behavior therapy, cost effectiveness, managed care, AODD (alcohol and other drug dependence) recovery, treatment program, evaluation, motivational interviewing, AOD (alcohol and other drug) abstinence, comparative study, literature review

## Abstract

Facilitating patients’ involvement with 12-step self-help organizations, such as Alcoholics Anonymous (AA) and Narcotics Anonymous (NA), is often a goal of substance abuse treatment. Twelve-step-facilitation (TSF) interventions have been found to be more effective than comparison treatments in increasing patients’ 12-step group involvement and in promoting abstinence. Future TSF evaluation research should address the effectiveness of incorporating TSF interventions with cognitive-behavioral treatment methods, the relative impact of brief versus extended TSF interventions, and the cost-effectiveness and health care cost-offset of TSF interventions within managed health care systems.

Although the United States has developed an extensive array of professional alcohol treatment services over the past 30 years, the peer-led, voluntary fellowship known as Alcoholics Anonymous (AA) continues to be the most widely accessed resource for people with alcohol problems (McCrady and Miller 1993). This article discusses the rationale for interventions that facilitate alcohol-dependent patients’ affiliations with AA and related mutual-help organizations (e.g., Narcotics Anonymous [NA]). The article also reviews recent research comparing those interventions with other treatment methods.

## Importance of 12-Step Group Affiliation in Alcohol Treatment

The rationale for facilitating patients’ involvement in 12-step self-help groups stems primarily from recent AA outcome research and from developments in the management and organization of health care in the United States. From the 1940s through the 1980s, most studies on AA did not directly evaluate AA’s effectiveness. Rather, researchers examined AA’s organizational structure and functioning; its history; and the ways in which AA participation changed members’ values, sense of identity, and spiritual outlook (see Kurtz 1993 for a review). The few AA outcome studies that were conducted typically did not study AA members over time or include non-AA members for comparison purposes, making conclusions about AA’s effectiveness tenuous. Given this limited empirical base, many clinicians and researchers doubted whether AA truly helped its members recover from alcohol dependence.

In the 1990s, the breadth and depth of AA research increased significantly, as evidenced by a National Institute on Alcohol Abuse and Alcoholism (NIAAA)-sponsored conference on AA (McCrady and Miller 1993), the International Collaborative Study of AA (Mäkelä et al. 1996), and other projects. Recent AA outcome research, which has demonstrated the benefits of treatments intended to facilitate AA involvement, as well as of AA involvement per se, has typically employed longitudinal designs (i.e., studied AA members over time), reliable measures, comparison groups and, in some cases, random assignment to conditions. The improved methodological quality of AA research has reduced skepticism in the treatment community about AA’s effectiveness and has increased clinicians’ interest in facilitating connections between substance abuse treatment and 12-step self-help groups.

The other major factor that has enhanced interest in 12-step facilitation (TSF) interventions is the growth of managed health care. In both the public and private sectors, managed care has reduced the length and intensity of professional addiction treatment services (Humphreys et al. 1997) and increased the pressure for cost-effective care. Because managed care has reduced the amount of time available for practitioners to work with patients, clinicians are increasingly interested in facilitating patient involvement in self-help groups as an inexpensive way to achieve and maintain treatment gains. Three recent studies (Tonigan et al. in press; Humphreys et al. 1999; McCrady et al. in press) have evaluated the effectiveness of such efforts.

## Recent Evaluations of TSF Interventions

One large study, known as Project MATCH, compared a TSF intervention with cognitive-behavioral (CB) therapy and motivational enhancement therapy (MET) among 1,726 patients (76 percent male) diagnosed with either alcohol abuse or dependence, including 774 inpatients who were beginning outpatient aftercare and 952 patients receiving outpatient care as their primary treatment (Project MATCH Research Group 1997, 1998). CB therapy focuses on teaching coping skills to reduce alcohol use (i.e., patients who use alcohol to cope with stress learn and practice alternative coping methods). In contrast, MET employs motivational strategies to mobilize patients’ internal resources for change.

The TSF intervention in the Project MATCH study was a form of one-on-one professional counseling explicitly designed to work synergistically with AA and other 12-step groups (Nowinski et al. 1992). Consistent with AA’s philosophy, TSF therapists presented alcohol dependence as a disease with spiritual, emotional, and physical components and emphasized that the disease could be arrested but not cured through permanent abstinence from alcohol. Also consistent with AA’s approach, patients were strongly urged, but neither ordered nor forced, to attend AA meetings and to maintain a journal describing their reactions to the meetings. The textbox above lists the major goals of the 12-session TSF intervention employed in Project MATCH.

**Major Goals of 12-Step Facilitation Therapy in Project Match**

***Acceptance***

- Acceptance by patients that they suffer from the chronic and progressive illness of alcoholism
- Acceptance by patients that they have lost the ability to control their drinking
- Acceptance by patients that because there is no effective cure for alcoholism, the only viable alternative is complete abstinence from the use of alcohol

***Surrender***

- Acknowledgment on the part of the patient that hope for recovery (i.e., sustained sobriety) exists, but only by accepting the reality of loss of control and by having faith that some higher power can help the patient, whose own willpower has been defeated by alcoholism
- Acknowledgment by the patient that the fellowship of Alcoholics Anonymous (AA) has helped millions of alcoholics sustain their sobriety and that the patient’s best chances for success are to follow the AA path

SOURCE: Adapted from Nowinski et al. 1992.

At both 1- and 3-year followups, patients in all three conditions (i.e., CB therapy, MET, and TSF therapy) had improved significantly on drinking-related (e.g., number of drinks per day and drinking consequences), psychological (e.g., depressive symptoms), and life-functioning (e.g., days of employment) outcomes. As predicted, TSF therapy was significantly more effective than either CB therapy or MET in increasing AA involvement, as indicated by the frequency of such patient behaviors as attending meetings, having and serving as a sponsor, following the 12 steps, and considering oneself an AA member (Tonigan et al. in press). In addition, TSF therapy was more effective than the other two treatments in promoting abstinence. For example, at the 3-year followup, 36 percent of TSF patients in the outpatient group reported being abstinent for the previous 3 months, compared with about 25 percent of outpatients in the CB therapy and MET treatment conditions. This result is consistent with the goals of TSF therapy and with AA, neither of which views moderate drinking as an acceptable or attainable goal for alcohol-dependent people.

Some Project MATCH participants who had received MET or CB therapy attended AA on their own, even though AA was not a focus of their treatment. The Project MATCH team examined whether those patients benefited less from AA affiliation than did those patients who had received TSF therapy (Tonigan et al. in press). Among the inpatient (aftercare) group, the positive relationship between AA attendance and more abstinent days and fewer drinks on drinking days was similar for all three treatment conditions. However, among the outpatients who received CB therapy, AA attendance was positively related to drinking in the first 6 months of the study (i.e., the patients in this group who attended the most AA meetings consumed the most alcohol). This result was not found at the 1-year followup and is the only exception to the general pattern of AA involvement associated with decreased drinking.

Concurrent with Project MATCH, the Department of Veterans Affairs (VA) conducted a nationwide treatment evaluation with more than 3,000 male substance abuse inpatients, who provided data when they entered treatment, when they were discharged, and 1 year later. (For an overview, see Moos and colleagues 1999; for results related to self-help groups, see Humphreys and colleagues 1999). Results presented here focus on the subset of inpatients treated in 21- to 28-day programs that exemplified either a 12-step approach or a CB approach.

The VA study included five 12-step-oriented inpatient programs, which emphasized treatment activities such as attending 12-step group meetings in the community and onsite, following the 12 steps, reading the *Big Book* (the primary text of AA, which presents AA’s philosophy mainly through narratives of members’ life stories) and other AA/NA literature, and accepting the identity of alcoholic/addict. Staff members in these programs strongly endorsed the disease model of addiction and reported spending most of their time on 12-step treatment activities. The study’s five CB treatment programs required that patients participate in relapse prevention groups, cognitive skills training, and cognitive-behavioral group therapy. Analysis of program schedules showed that CB programs spent less than 5 percent of treatment time on activities based on 12-step principles.

The study participants (2,045 men) were treated in either a 12-step-oriented or a CB inpatient program and were followed up 1 year after discharge. Thirty-six percent of the participants had either an alcohol abuse or dependence diagnosis only, 13 percent had only a drug abuse or dependence diagnosis, and 51 percent had both drug and alcohol diagnoses.

**Figure:**
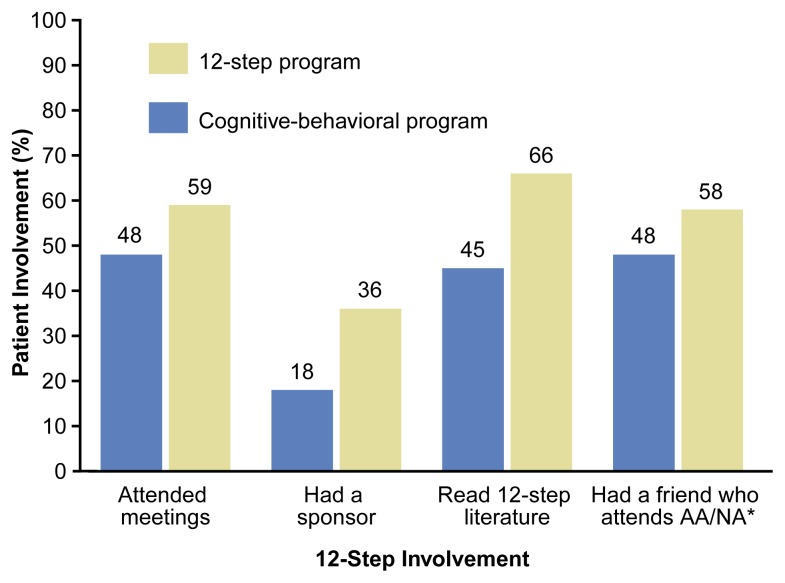
Posttreatment 12-step self-help group involvement of 2,045 substance-dependent veterans treated by 12-step or cognitive-behavioral programs. *AA/NA = Alcoholics Anonymous/Narcotics Anonymous. NOTE: Involvement was measured 1 year after discharge by patient reports of activities in the past 3 months. SOURCE: Humphreys et al. 1999.

Before treatment, patients in 12-step-oriented and CB programs had similar levels of self-help group involvement. However, 1 year after treatment, the patients who had received 12-step-oriented treatment reported substantially greater involvement in AA/NA during the previous 3 months than did patients who had received CB treatment. Patients in 12-step-oriented treatment were significantly more likely than those in CB treatment to have attended 12-step meetings, to have a sponsor, to have read 12-step literature, and to have a friend who attends meetings (see figure, above), indicating that 12-step-oriented treatment programs were more effective than CB programs at increasing affiliation with 12-step self-help groups.

The results of the VA study closely parallel those of Project MATCH. In both 12-step-oriented and CB treatment, patients experienced substantial reductions in substance use, substance abuse-related problems, psychological problems, criminal behavior, and unemployment (Moos et al. 1999). The reductions were comparable across treatments, with the only notable exception again being that 12-step-oriented treatment was more effective in promoting abstinence. For example, 1 year after treatment, 45 percent of patients treated in 12-step-oriented programs reported abstinence from alcohol and other drugs during the previous 3 months, compared with 36 percent of patients treated in CB programs.

Like Project MATCH, the VA study also found some evidence suggesting that 12-step self-help group involvement after treatment may be more beneficial for patients who have received 12-step-oriented treatment, compared with patients who have received CB treatment. Among patients who received 12-step-oriented treatment, abstinence rates 1 year after treatment varied from 19 percent for patients with low involvement with self-help groups after treatment to 75 percent for patients with high involvement. Among patients who received CB treatment, abstinence rates ranged from 25 percent for patients with low involvement in self-help groups after treatment to 65 percent for patients with high involvement. This difference was statistically significant, indicating that 12-step self-help group involvement after treatment seems to benefit patients treated in 12-step-oriented programs more than it does patients treated in CB programs (Humphreys et al. 1999).

A third study comparing a TSF intervention with other treatment focused on 60 heterosexual couples in which the men were diagnosed with either alcohol abuse or dependence and their female partners were not. As part of a larger study, the couples received either outpatient, alcohol-focused, CB marital therapy only (MT-only), or the MT intervention supplemented with counseling designed to facilitate involvement in 12-step self-help groups (MT+TSF) (McCrady et al. in press). The TSF intervention introduced patients to the philosophies and methods of AA and Al-Anon,^1^ helped patients identify meetings at convenient locations, set goals for meeting attendance, and encouraged patients to choose a sponsor and to follow the 12 steps. The TSF therapists in this study assigned patients AA- or Al-Anon-related homework, as appropriate, and emphasized parallels between the initial CB therapy and AA techniques.

Patients in both the MT-only condition and the MT+TSF condition attended an average of approximately 10 outpatient sessions. Patients in the MT+TSF condition were more likely than those in the MT-only group to attend 12-step group meetings during treatment. Eighty-two percent of MT+ TSF patients attended AA meetings, and 58 percent attended Al-Anon meetings, compared with attendance rates of 15 percent and 5 percent, respectively, among patients in the MT-only group. At 6 months after treatment, however, about half the patients in both groups were either abstinent or engaged in nonproblem drinking, indicating that supplementing CB marital therapy with TSF did not appear to enhance the effectiveness of the intervention.

## Conclusions from Recent Research

When analyzed together, the results from Project MATCH, the VA study, and the couples study (reported by McCrady and colleagues [in press]) support the following three conclusions about TSF interventions:

- Professional interventions can powerfully influence substance-abuse patients’ level of affiliation with 12-step self-help groups. All three studies found that when providers encouraged patients to attend meetings, follow the 12 steps, choose a sponsor, and engage in other AA- or NA-related behaviors, patients were substantially more likely to be involved in self-help groups during and after treatment. Hence, involvement in 12-step self-help groups is not (as is sometimes claimed) simply an indicator of patient motivation; rather, it is a behavior that clinicians can influence. The optimistic implication is that by incorporating 12-step facilitation into treatment, providers can increase the likelihood that patients will continue to improve even after professional treatment has ended.
- Twelve-step-oriented treatments that increase involvement in 12-step self-help groups generally produce outcomes comparable with those of CB treatments and are somewhat more effective in promoting abstinence. Project MATCH and the VA study provide strong evidence that 12-step-oriented treatments are as effective as more heavily researched CB interventions. This finding increases confidence in the U.S. substance abuse treatment system, because many of the prevalent treatment modalities in the United States draw heavily on 12-step principles (Borkman et al. 1998).
- Combining CB treatments with AA/NA affiliation may be less helpful to patients than combining 12-step treatment with AA/NA affiliation. None of the studies discussed were specifically designed to address whether positive results are maximized when treatment and self-help groups operate from a similar orientation; thus, this statement is more a working hypothesis than a firm conclusion. Perhaps patients who hear contradictory messages about substance abuse from treatment providers and self-help groups have more difficulty understanding and practicing treatment regimens. To evaluate this possibility, CB treatment providers could develop interventions that promote involvement in CB-oriented self-help groups (e.g., SMART Recovery and Moderation Management both draw heavily on CB intervention research and theory) and determine whether such interventions enhance outcomes more than do TSF interventions. An alternative explanation for the findings to date is that the subset of patients who switch from CB treatment to 12-step self-help groups do so because they find CB treatment ineffective and seek an alternative that is better matched to their needs.

This hypothesis would be an excellent one for future research. Other potential research directions are presented in the following section.

## Directions for Future Research

The studies reviewed above clearly demonstrate that TSF interventions that engage patients for a significant period (e.g., 10 to 12 sessions of outpatient care, as in Project MATCH and the couples study [McCrady et al. in press], or 21 to 28 days of 12-step-oriented inpatient care, as in the VA study), can sharply increase the likelihood that patients will attend AA/NA. However, many patients in primary care and addiction treatment settings are not treated for extended periods. To make TSF interventions more useful in practice, researchers and clinicians should develop and evaluate brief TSF interventions. In one promising study, Sisson and Mallams (1981) randomly assigned alcohol outpatients to a “simple” or “enhanced” TSF intervention. In the simple condition, a therapist suggested that the patient attend AA or Al-Anon and provided a printed list of meeting times and locations. In the enhanced condition, the therapist supplemented the aforementioned intervention with an in-session telephone call to a current member of AA or Al-Anon, who talked to the patient briefly and arranged to attend a meeting with him or her. The 12-step group member contacted the patient with a reminder telephone call the night before the meeting, drove the patient to the meeting, and let the patient’s therapist know on the following day whether the patient had attended. During the month following the intervention, 100 percent of patients in the enhanced TSF group attended at least one meeting (average 2.3 meetings), compared with zero-percent meeting attendance among patients in the simple TSF group. Although the study only followed patients for 1 month, the results suggest that a fairly brief intervention (i.e., the enhanced TSF intervention) can have a significant impact.

A related research priority is to evaluate whether TSF interventions can promote AA/NA affiliation in the long term to the same extent that they increase meeting attendance in the short term. The fact that patients’ attendance at meetings increases significantly during and in the months following a TSF intervention does not guarantee that patients will become active affiliates of AA who continue to identify with the organization, sponsor newcomers, celebrate sober anniversaries, and read 12-step materials. To examine this important issue, researchers must extend their followups beyond the common 6- and 12-month periods and continue to examine a broad range of AA affiliation indices (i.e., indicators other than meeting attendance).

A third key priority for future research is to more fully understand the health care cost offset and cost-effectiveness of TSF interventions. One study of 227 industrial plant workers with alcohol problems (i.e., employees averaging 6 drinks per day and 20 drinking days per month) found that patients assigned directly to AA had similar work-related outcomes but more relapses than did patients assigned to inpatient treatment followed by AA. At the same time, patients initially assigned to AA had 10-percent lower alcohol-related health care costs over a 2-year period than did patients initially assigned to inpatient treatment (Walsh et al. 1991). Similarly, in a study of patients with serious alcohol problems (e.g., alcohol dependence symptoms such as shakes and hallucinations), those who sought outpatient care and those who sought AA experienced similar improvement over a 3-year period on a variety of measures. The AA-seeking group, however, had 45 percent lower (approximately $1,800 per person) alcohol-related-treatment costs (e.g., costs of counseling and detoxification) over the 3-year period compared with the outpatient treatment-seeking group (Humphreys and Moos 1996). Both studies suggest that when patients with alcohol problems are connected to the AA network, they lower their reliance on professional health care. A priority for future research should be to evaluate whether this reduction in health care costs is sufficient to offset the cost of TSF interventions. Findings from that research could inform and improve health care policy. For example, if clinicians could differentiate patients who were likely to recover with a brief TSF intervention from patients who required extensive professional treatment, the treatment system could become more cost efficient without compromising patient outcomes.

Thanks in part to support from Federal agencies, research on TSF interventions and AA has expanded significantly in recent years. The next decade will offer researchers and clinicians the opportunity to build on this foundation. AA and other self-help organizations are the most commonly sought resource for substance abuse problems in the United States, currently reaching more than 1 million Americans (McCrady and Miller 1993); determining how treatment providers and self-help groups can more effectively collaborate in our system of formal and informal care will benefit many substance-dependent Americans.

## Footnotes

1 Al-Anon is a self-help organization for spouses and significant others in alcohol-dependent people’s lives.
